# Multimodality imaging in the diagnosis of biliary rhabdomyosarcoma in a young child: a case report

**DOI:** 10.3389/fped.2026.1852972

**Published:** 2026-07-16

**Authors:** Tianjiao Huang, Peng Wang, Danqing Huang, Jun Chen, Wentao Kong, Lichun Hua

**Affiliations:** 1 Department of Ultrasound, Nanjing Drum Tower Hospital, Affiliated Hospital of Medical School, Nanjing University, Nanjing, China; 2 Department of Ultrasound, Tangdu Hospital, Air Force Medical University, Xi'an, Shaanxi Province, China; 3 Department of Ultrasound, Children’s Hospital of Nanjing Medical University, Nanjing, Jiangsu, China

**Keywords:** biliary rhabdomyosarcoma, case report, multimodality imaging, pediatric oncology, pediatric surgery

## Abstract

**Background:**

Rhabdomyosarcoma (RMS) is the most common soft tissue sarcoma in children, but biliary involvement is extremely rare, accounting for approximately 0.5% of all pediatric RMS cases.

**Case presentation:**

We report a 2-year-11-month-old girl presenting with obstructive jaundice, whose condition was initially misdiagnosed as choledochal cysts. Multimodality imaging—ultrasound, contrast-enhanced computed tomography (CT), magnetic resonance imaging (MRI)/ magnetic resonance cholangiopancreatography (MRCP), and positron emission tomography (PET)-CT—revealed a solid hilar mass with biliary dilation. This integrated imaging approach guided subsequent endoscopic retrograde cholangiopancreatography (ERCP) with stenting and open biopsy. Histopathology confirmed embryonal RMS (MyoD1+/Myogenin+). The patient was staged as intermediate-risk (stage III) and received vincristine, actinomycin D, and cyclophosphamide (VAC) chemotherapy.

**Conclusion:**

Multimodality imaging is critical for timely diagnosis and staging of biliary RMS, enabling accurate biopsy and guiding multidisciplinary management.

## Introduction

RMS is the fourth most common solid tumor in children and the third most common extracranial solid tumor, following neuroblastoma and Wilms tumor. Biliary RMS is a rare subset, accounting for approximately 0.5% of all pediatric RMS cases (1). The two primary histological subtypes in children are embryonal RMS and alveolar RMS (2).

Imaging plays a pivotal role in the diagnosis of this disease. Ultrasonography typically reveals biliary duct dilation and an intraluminal mass, while MRCP further delineates biliary involvement. Treatment requires a multimodal approach including surgery, chemotherapy, and radiotherapy. Complete surgical resection is often challenging due to the tumor's location. Although prognosis has historically been poor, early diagnosis and aggressive treatment can significantly improve outcomes. Approximately 100 cases have been reported globally. This case highlights the role of multimodality imaging in the diagnosis and staging of this rare entity.

## Case description

A 2-year-11-month-old girl was admitted to a local hospital in July 2024 with a 1-week history of dark urine and cutaneous jaundice. Liver biochemical tests revealed abnormal hepatic function, and abdominal ultrasonography demonstrated dilatation of both intrahepatic and extrahepatic bile ducts, consistent with biliary obstruction. Initial MRCP suggested biliary sludge filling the extrahepatic bile ducts, leading to an initial misdiagnosis of biliary sludge lesion. The patient was thereafter transferred to our institution for definitive diagnosis and further management.

Upon admission, relevant examinations were performed. Biochemical tests revealed: total bilirubin 187.9 μmol/L, direct bilirubin 185.2 μmol/L, alanine aminotransferase 103 U/L, aspartate aminotransferase 99 U/L, alkaline phosphatase 519 U/L, and gamma-glutamyl transferase 160 U/L. Urinalysis showed positive urobilinogen and negative bilirubin. The tumor marker carbohydrate antigen 19-9 (CA19-9) was elevated at 377 U/mL.

Ultrasonography revealed a 25 mm × 13 mm × 23 mm hypoechoic solid mass located in the porta hepatis, anterior to the portal vein, with ill-defined borders and detectable intratumoral blood flow signals on color Doppler (Figures 1A,B). The intrahepatic bile ducts were tortuous and dilated (Figure 1C). Diffuse wall thickening (>1.5 mm) with increased echogenicity involved the common bile duct and portions of the intrahepatic bile ducts. No abnormally enlarged lymph nodes or portal vein emboli were observed.

**Figure 1:**
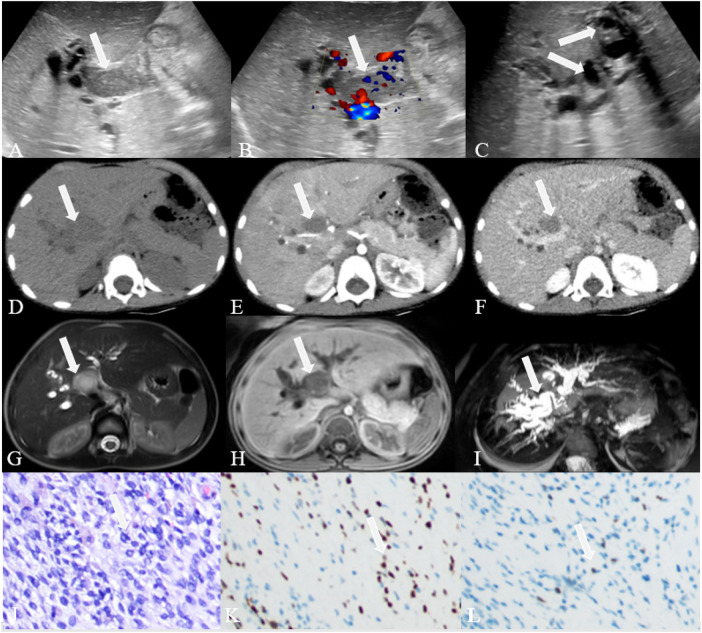
Multimodality imaging and pathological findings of pediatric hilar biliary embryonal rhabdomyosarcoma. **(A–C)** Abdominal ultrasound; **(D–F)** Contrast-enhanced computed tomography; **(G–I)** Magnetic resonance imaging and MRCP; **(J–L)** Histopathology and MyoD1/myogenin immunohistochemical staining. White arrows mark the primary biliary tumor lesion in each panel.

Contrast-enhanced CT showed a round, ill-defined, low-density mass in the porta hepatis. After contrast administration, the lesion's periphery showed marked enhancement in the arterial phase, with moderate central enhancement. The left and right hepatic arteries were seen coursing adjacent to the lesion with no evidence of vascular encasement (Figures 1D,E). Enhancement persisted into the venous and delayed phases but remained lower than that of the adjacent liver parenchyma (Figure 1F).

MRI demonstrated an irregular lesion in the hepatic hilum with slightly prolonged T1-weighted signals and prolonged T2-weighted signals, heterogeneous intensity, and indistinct margins. Diffusion-weighted imaging (DWI) presented obvious hyperintense signals, and the corresponding apparent diffusion coefficient (ADC) map displayed hypointense signals, suggestive of restricted water diffusion within the tumor. A tortuous cystic duct was observed within the lesion. Post-contrast images showed heterogeneous enhancement (Figure 1G). MRCP revealed marked dilation of the left and right hepatic ducts and intrahepatic bile ducts, while the common hepatic duct and proximal common bile duct were poorly visualized; the distal common bile duct and main pancreatic duct were not dilated (Figures 1H,I).

PET-CT revealed a round, fluorodeoxyglucose (FDG)-avid, cystic-solid soft tissue lesion in the common bile duct at the hepatic hilum, with heterogeneous radiotracer uptake and a maximum standardized uptake value (SUVmax) of 3.9. The lesion had poorly defined borders and involved the adjacent left and right hepatic ducts and their confluence. No FDG-avid lymphadenopathy or abnormal metabolic foci were observed elsewhere.

To alleviate biliary tract obstruction, endoscopic retrograde cholangiopancreatography (ERCP) with biliary stent placement was performed in July 2024. Dynamic changes in liver biochemical parameters before and after ERCP stenting were summarized in Table 1. Subsequently, open surgical biopsy of the lesion was conducted under general anesthesia. Intraoperative exploration revealed hepatic biliary stasis, marked gallbladder distension, and severe thickening of the common bile duct wall. After adhesiolysis, an intraluminal tumor mass was exposed, and partial tissue resection was performed for histopathological testing. Hematoxylin and eosin (H&E) staining demonstrated diffusely proliferative tumor cells with oval or short spindle morphology, hyperchromatic nuclei and scant cytoplasm (Figure 1J). Immunohistochemical staining revealed diffuse positive expression of vimentin, desmin, MyoD1, SMA, HIF-1*α* and CD99; focal positive staining for myogenin; partial positivity for CD34; and negative immunoreactivity for S100, LCA and ALK. The Ki-67 proliferation index reached 70% (Figures 1K,L). These results verified the pathological diagnosis of embryonal RMS. According to the Intergroup Rhabdomyosarcoma Study (IRS) surgical staging system, the patient was classified as stage III, corresponding to the intermediate-risk group.

**Table 1:** Comparison of liver function tests before and after ERCP biliary stenting.

Liver function parameters	Before ERCP stenting	After ERCP stenting
Alanine aminotransferase U/L	132	76
Aspartate aminotransferase U/L	91	83
Alkaline phosphatase U/L	700	357
Gamma-glutamyl transferase U/L	260	172
Albumin g/L	39.8	31.9
Total bilirubin μmol/L	103.6	41.8
Conjugated bilirubin μmol/L	102.6	40.9

Considering the tumor's hepatic hilar location, ill-circumscribed margins, dense adhesions to surrounding bile ducts and blood vessels, together with the stage III intermediate-risk RMS stratification, the multidisciplinary team decided to initiate neoadjuvant chemotherapy. This treatment strategy was selected per the risk-adapted clinical guidelines for pediatric RMS published by the Children's Oncology Group (COG), with the goals of reducing tumor burden and mitigating perioperative complications. The patient received VAC combination chemotherapy from August to December 2024 and was discharged with stable vital signs. After four cycles of neoadjuvant chemotherapy, follow-up MRI demonstrated remarkable volumetric regression of the biliary tumor (Figures 2A,B). After multidisciplinary consultation among the hepatobiliary surgical team and full communication with the patient's guardians, radical resection of the hilar biliary tumor combined with hepaticojejunostomy and choledochojejunostomy was performed on December 20, 2024. Postoperative MRI showed peri-Glibissonian sheath edema without residual tumor (Figure 2C). Postoperatively, serum CA19-9 decreased to 31.2 U/mL, and postoperative pathological examination confirmed negative surgical resection margins. Sequential adjuvant VAC chemotherapy was restarted on March 7, 2025. As of May 8, 2026, long-term follow-up MRI showed alleviated peri-Glibissonian sheath edema without any signs of local tumor recurrence or distant metastasis (Figure 2D), and the patient has achieved sustained favorable clinical outcomes. The complete chronological diagnostic and therapeutic course of this patient is systematically summarized in the timeline diagram (Figure 3).

**Figure 2:**
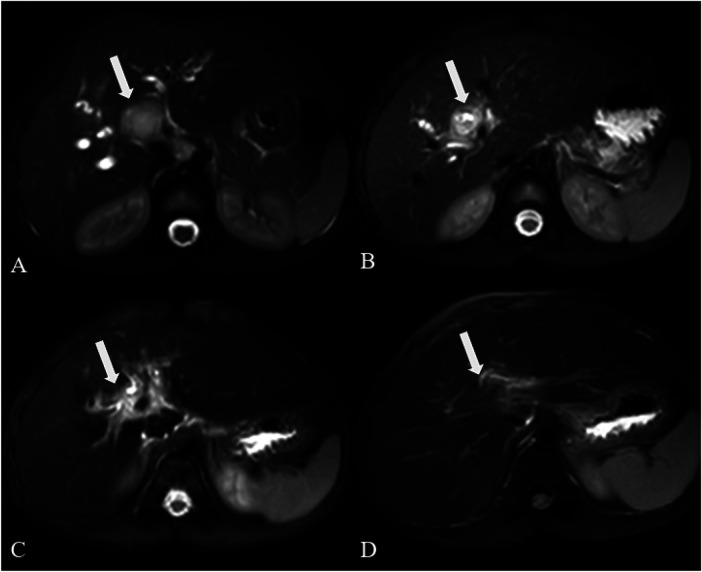
Dynamic MRI follow-up images of liver lesions during the treatment cycle. **(A)** Baseline MRI of the patient showing the primary tumor. **(B)** Post-chemotherapy MRI demonstrating marked tumor regression. **(C)** MRI after surgical resection presenting peri-Glissonian sheath edema. **(D)** The latest follow-up MRI revealing alleviated peri-Glissonian sheath edema without evidence of tumor recurrence.

**Figure 3:**
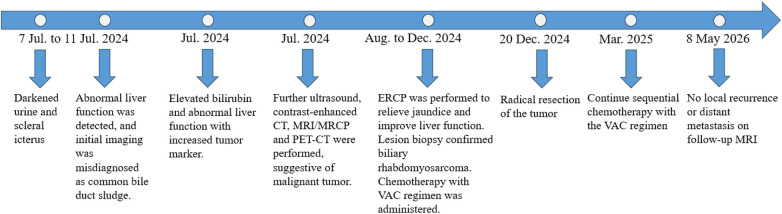
Timeline of diagnosis and treatment for pediatric biliary rhabdomyosarcoma.

## Discussion

RMS is the most common soft tissue sarcoma in children, accounting for approximately 5% of all childhood malignancies. However, biliary RMS represents only 0.5% of pediatric RMS cases (3). This tumor originates from the bile duct wall, most frequently in the common bile duct, followed by the hepatic duct and intrahepatic bile ducts. The median age at presentation is approximately 3 years, with a slight male predominance (4).

RMS arises from undifferentiated mesenchyme and exhibits features of embryonic skeletal muscle differentiation. It is composed of primitive, poorly differentiated mesenchymal cells. Embryonal RMS specifically expresses MyoD1 and myogenin, whereas myogenin expression in alveolar RMS is typically stronger and more diffuse. In our patient, tumor cells expressed MyoD1 and a small amount of myogenin, supporting the diagnosis of embryonal RMS.

The diagnosis of biliary RMS is challenging due to its rarity. Common clinical presentations include jaundice, dark urine, pale stools, and abdominal pain. Biochemical findings typically show moderate to severe direct hyperbilirubinemia, accompanied by mild elevations in transaminases and alkaline phosphatase (5).

Imaging is crucial for diagnosis. Ultrasonography, often the first-line modality due to its lack of radiation and real-time capability, typically reveals a hypoechoic or heterogeneously echogenic mass with internal blood flow. Large masses may compress the portal vein and cause intra- or extrahepatic bile duct dilation. Ultrasound is also useful for follow-up to monitor tumor size, recurrence, and lymph node metastasis (6). However, biliary RMS is frequently missed or misdiagnosed on ultrasound, as it can be mistaken for biliary stones or malformations—as occurred in the initial evaluation of this case. This may be due to insufficient recognition of key features: (1) an infiltrative hypoechoic hilar mass may be overlooked, and (2) malignant indicators such as bile duct wall thickening and abundant intratumoral blood flow (RI >0.5) may not be adequately appreciated. In children with obstructive jaundice, focused scanning of the hilar region with a high-frequency probe is essential. The combination of “bile duct dilation with wall thickening plus a hypervascular hilar mass” should raise strong suspicion for RMS.

CT and MRI serve as complementary modalities to assess tumor resectability. On MRI, biliary RMS typically appears as a mass originating from and involving the bile duct. It is usually hypointense to isointense on T1-weighted images and hyperintense or heterogeneously hyperintense on T2-weighted images, often due to central necrosis. Diffusion-weighted imaging shows marked restriction with low apparent diffusion coefficient (ADC) values. After contrast administration, the mass enhances heterogeneously. Hepatoblastoma may show similar imaging features, but a markedly elevated serum alpha-fetoprotein (AFP) level is a key distinguishing feature. In our patient, AFP was not elevated, leading the radiologists to favor biliary RMS.

When the mass presents with cystic and solid components secondary to hemorrhage and necrosis, differentiation from a choledochal cyst can be challenging (2). We systematically compared biliary RMS with other common etiologies of pediatric biliary obstruction. A choledochal cyst is a congenital biliary malformation appearing as a purely cystic lesion with no solid tissue or internal vascularity on imaging, in sharp contrast to the hypervascular solid mass characteristic of biliary RMS (3). Hepatoblastoma typically causes a substantial rise in serum AFP and frequently shows intratumoral calcification on CT, features that are absent in biliary RMS where AFP levels stay within normal range (4). Cholangiopathy mainly leads to bile duct wall thickening and stenosis without a distinct mass lesion, while inflammatory or infectious biliary obstruction is clinically accompanied by fever and abdominal colic, with imaging demonstrating bile duct wall edema and periductal fat infiltration instead of a malignant soft-tissue mass (7). ERCP with stent placement can effectively relieve biliary obstruction and help obtain tissue samples in these scenarios; nevertheless, open biopsy was performed in our patient to reach a definitive histopathological diagnosis (8).

Treatment for biliary RMS includes surgery, chemotherapy, and radiotherapy. Surgical resection depends on tumor size and location. Tumors confined to the primary site and measuring ≤5 cm are typically resected to achieve stage I. Larger tumors (postoperative stages II or III) generally require adjuvant chemotherapy (9). The VAC regimen (vincristine, actinomycin D, cyclophosphamide) is the standard chemotherapy backbone recommended by COG clinical guidelines, as used in this case (5). If necessary, ifosfamide and etoposide may be added. For patients with widespread metastasis at diagnosis, initial chemotherapy with or without radiotherapy may be used to facilitate subsequent surgery. In cases of intrahepatic biliary RMS with extensive liver metastasis but no extrahepatic spread, liver transplantation has been proposed as a potential curative option (10). For patients at high risk of surgical complications, pencil-beam scanning proton therapy may be a viable alternative (11).

One important limitation of this case report is the absence of PAX3/7-FOXO1 fusion gene testing. Due to limited residual biopsy specimens, molecular fusion detection could not be performed. Currently, FOXO1 fusion status is a core biomarker integrated into the COG risk stratification system to predict prognosis (12). Although MyoD1 and myogenin immunohistochemistry confirmed embryonal RMS histology, the missing fusion data prevents complete modern risk stratification assessment, which is acknowledged as a major limitation of this study.

## Conclusion

Biliary RMS is a rare pediatric malignancy presenting with obstructive jaundice. A multimodal imaging approach—ultrasound, CT, MRI, and ERCP with biopsy—is essential for timely diagnosis. Histopathological confirmation with MyoD1 and myogenin immunostaining is critical. Management combines chemotherapy (notably VAC) and surgery, with treatment tailored to stage and resectability. Multidisciplinary care has significantly improved outcomes.

## Data Availability

The original contributions presented in the study are included in the article/supplementary material, further inquiries can be directed to the corresponding author/s.
